# *GSTM1* and *GSTT1* Null Polymorphisms and Childhood Acute Leukemia Risk: Evidence from 26 Case-Control Studies

**DOI:** 10.1371/journal.pone.0078810

**Published:** 2013-10-23

**Authors:** Qiuqin Tang, Jing Li, Simin Zhang, Beilei Yuan, Hong Sun, Di Wu, Chuncheng Lu, Wei Wu, Yankai Xia, Hongjuan Ding, Lingqing Hu, Daozhen Chen, Jiahao Sha, Xinru Wang

**Affiliations:** 1 State Key Laboratory of Reproductive Medicine, Department of Obstetrics, Nanjing Maternity and Child Health Care Hospital Affiliated to Nanjing Medical University, Nanjing, Jiangsu, China; 2 State Key Laboratory of Reproductive Medicine, Institute of Toxicology, Nanjing Medical University, Nanjing, Jiangsu, China; 3 Key Laboratory of Modern Toxicology of Ministry of Education, School of Public Health, Nanjing Medical University, Nanjing, Jiangsu, China; 4 Department of Public Health, Xuzhou Medical College, Xuzhou, Jiangsu, China; 5 Department of Microbial and Molecular SystemsLeuven, Leuven, Belgium; 6 State Key Laboratory of Reproductive Medicine, Wuxi Maternity and Child Health Care Hospital Affiliated to Nanjing Medical University, Wuxi, Jiangsu, China; Baylor College of Medicine, United States of America

## Abstract

Several molecular epidemiological studies have been conducted to examine the association between glutathione S-transferase mu-1 (*GSTM1*) and glutathione S-transferase theta-1 (*GSTT1*) null polymorphisms and childhood acute leukemia; however, the conclusions remain controversial. We performed an extensive meta-analysis on 26 published case-control studies with a total of 3252 cases and 5024 controls. Crude odds ratios (ORs) with 95% confidence interval were used to assess the strength of association between childhood acute leukemia risk and polymorphisms of *GSTM1* and *GSTT1*. With respect to *GSTM1* polymorphism, significantly increased risk of childhood acute leukemia was observed in the overall analysis (OR = 1.30; 95%CI, 1.11-1.51). Furthermore, a stratification analysis showed that the risk of *GSTM1* polymorphism are associated with childhood acute leukemia in group of Asians (OR = 1.94; 95%CI, 1.53-2.46), Blacks (OR = 1.76; 95%CI, 1.07-2.91), ALL (OR = 1.33; 95%CI, 1.13-1.58), ‘< 100 cases and <100 controls’ (OR = 1.79; 95%CI, 1.21-2.64), ‘≥ 100 cases and ≥ 100 controls’ (OR = 1.25; 95%CI, 1.02-1.52), and population-based control source (OR = 1.40; 95%CI, 1.15-1.69). With respect to *GSTT1* polymorphism, significant association with childhood acute leukemia risk was only found in subgroup of Asian. This meta-analysis supports that *GSTM1* null polymorphism is capable of causing childhood acute leukemia susceptibility.

## Introduction

Leukemia is the most common form of cancer in childhood accounting for approximately one third of all childhood cancers [1]; which is a heterogeneous disease lacking a high penetrant germ line-inherited predisposition, except for rare cases with genetic instability or immunodeficiency syndromes. Although overall long-term disease-free survival has been improved to higher than 70% with modern chemotherapy [2], the etiology of this disease remains unknown due to the probable multifactorial mechanisms of pathogenesis. However, molecular epidemiologic case-control studies suggest that children harboring null genotype of the glutathione S-transferase mu-1 (*GSTM1*) and glutathione S-transferase theta-1 (*GSTT1*) genes might have an increased risk of the development of childhood acute leukemia. 

The GSTM1 and GSTT1 are phase II metabolic enzymes have the ablity to detoxify a wide variety of electrophilic compounds including the activated carcinogens. Human glutathione S-transferases are divided into eight distinct classes as alpha, kappa, mu, omega, pi, sigma, theta, and zeta based on amino acid sequence similarity and antibody cross-reactivity [3,4]. The mu class of GSTs, encoded by the *GSTM1* gene, is found on the chromosome 1p13.3 [5]. The theta class of GSTs, encoded by the *GSTT1* gene, is locate on the chromosome 22q11.23 [6]. Homozygotes for null alleles (deletion) of *GSTM1* and *GSTT1* have absent activity of the respective enzyme. DNA-adduct formation and rates of somatic mutation have been reported to be increased in carriers of null alleles [7]. Individuals with homozygous deletion polymorphism are considered to be at increased risk for malignancies due to reduced efficiency in protection against environmental carcinogens [8,9]. An increased frequency of *GSTM1* and *GSTT1* null genotypes has been associated with several types of malignancies, including stomach cancer [10], lung cancer [11], pituitary adenomas [12], bladder cancer [13], prostate cancer [14], cervical cancer [15], and acute leukemia [16]. 

GST polymorphisms were first reported as risk factors for childhood acute leukemia in 1997 [17]. Since then, a number of molecular epidemiological studies have been conducted to examine the association between polymorphisms within the *GSTM1, GSTT1* gene and childhood acute leukemia in diverse populations [18–33]. However, the results were inconsistent or even contradictory (Table 1 and Table 2). Individual studies are typically underpowered to detect associations with *GSTM1* and *GSTT1* of small effect sizes. To estimate the effect of *GSTM1* and *GSTT1* polymorphisms on the childhood acute leukemia, as well as to quantify the potential between-study heterogeneity, we conducted a meta-analysis on 26 published case-control studies with a total of 3252 cases and 5024 controls.

**Table 1:** Main characteristics of all studies of *GSTM1* genotypes included in the meta-analysis.

**First author**	**Year**	**Country**	**Ethnicity**	**Subtype of acute leukemia**	**Case**	**Age** ^a^	**Sex** ^b^	**Control**	**Age** ^a^	**Sex** ^b^	**Case**		**Control**		**Control source**
											**Present**	**Null**	**Present**	**Null**	
Chen CL	1997	USA	White	ALL	163	N/A	85/78	213	18-60	111/102	73 (44.8)	90 (55.2)	99 (46.5)	114 (53.5)	Population
Chen CL	1997	USA	Black	ALL	34	N/A	22/12	203	18-60	103/100	20 (58.8)	14 (41.2)	147 (72.4)	56 (27.6)	Population
Krajinovic M	1999	Canada	White	ALL	174	1-21	N/A	304	N/A	N/A	61 (35.1)	113 (64.9)	148 (48.7)	156 (51.3)	Population
Saadat I	2000	Iran	Asian	ALL	38	3-13	26/12	75	3-13	48/27	17 (44.7)	21 (55.3)	51 (68.0)	24 (32.0)	Population
Woo MH	2000	USA	White	AML	40	N/A	N/A	160	N/A	N/A	25 (62.5)	15 (37.5)	69 (43.1)	91 (56.9)	Hospital
Woo MH	2000	USA	Black	AML	7	N/A	N/A	38	N/A	N/A	2 (28.6)	5 (71.4)	24 (63.2)	14 (36.8)	Hospital
Woo MH	2000	USA	White	AML	6	N/A	N/A	44	N/A	N/A	2 (33.3)	4 (66.7)	25 (56.8)	19 (43.2)	Hospital
Davies SM	2000	USA	White	AML	232	N/A	N/A	153	N/A	N/A	168 (72.4)	64 (27.6)	106 (69.3)	47 (30.7)	Population
Davies SM	2002	USA	White	ALL	616	N/A	N/A	532	N/A	N/A	285 (46.3)	331 (53.7)	246 (46.2)	286 (53.8)	Hospital
Davies SM	2002	USA	Black	ALL	35	N/A	N/A	201	N/A	N/A	21 (60.0)	14 (40.0)	137 (68.2)	64 (31.8)	Hospital
Alves S	2002	Portugal	White	ALL	47	N/A	N/A	102	N/A	N/A	15 (31.9)	32 (68.1)	52 (51.0)	50 (49.0)	Population
Krajinovic M	2002	Canada	White	ALL	269	N/A	N/A	301	N/A	N/A	118 (43.9)	151 (56.1)	160 (53.2)	141 (46.8)	Hospital
Balta G	2003	Turkey	White	ALL	139	0.58-17	96/48	185	0.58-17	120/65	62 (44.6)	77 (55.4)	84 (45.4)	101 (54.6)	Population
Balta G	2003	Turkey	White	ANLL	31	1-17	19/14	185	0.58-17	120/65	12 (38.7)	19 (61.3)	84 (45.4)	101 (54.6)	Population
Barnettee P	2004	USA	White	ALL	94	N/A	N/A	326	N/A	N/A	46 (48.9)	48 (51.1)	143 (43.9)	183 (56.1)	Population
Canalle R	2004	Brazil	White	ALL	113	0.22-18	73/40	221	18-58	159-62	65 (57.5)	48 (42.5)	120 (54.3)	101 (45.7)	Population
Joseph T	2004	India	Asian	ALL	118	0-14	77/41	118	0-14	77/41	70 (59.3)	48 (40.7)	89 (75.4)	29 (24.6)	Hospital
Wang J	2004	China	Asian	ALL	67	N/A	N/A	146	N/A	N/A	16 (23.9)	51 (76.1)	69 (47.3)	77 (52.7)	Population
Wang J	2004	China	Asian	AML	32	N/A	N/A	146	N/A	N/A	9 (28.1)	23 (71.9)	69 (47.3)	77 (52.7)	Population
Clavel J	2005	France	White	ALL	191	< 15	N/A	105	N/A	57/48	97 (50.8)	94 (49.2)	55 (52.4)	50 (47.6)	Hospital
Clavel J	2005	France	White	ANLL	28	< 15	N/A	105	N/A	57/48	14 (50.0)	14 (50.0)	55 (52.4)	50 (47.6)	Hospital
Pakakasama S	2005	Thailand	Asian	ALL	107	0.83-14.75	62/45	320	N/A	165/155	31 (29.0)	76 (71.0)	129 (40.3)	191 (59.7)	Population
Aydin-Sayitoglu M	2006	Turkey	White	ALL	119	N/A	N/A	140	16-59	73/67	41 (34.5)	78 (65.5)	63 (45.0)	77 (55.0)	Population
Aydin-Sayitoglu M	2006	Turkey	White	AML	44	N/A	N/A	140	16-59	73/67	16 (36.4)	28 (63.6)	63 (45.0)	77 (55.0)	Population
Pigullo S	2007	Italy	White	ALL	323	< 18	N/A	384	< 18	N/A	171 (52.9)	152 (47.1)	184 (47.9)	200 (52.1)	Hospital
Chan JY	2011	Indonesia	Asian	ALL	185	0.03-14	107/78	177	N/A	104/73	43 (23.2)	142 (76.8)	55 (31.1)	122 (68.9)	Population

^a^ range of age (year); ^b^ male/female; ALL, acute lymphoblastic leukemia; AML, acute myeloid leukemia; ANLL, acute non-lymphoblastic leukemia; NA, not available.

**Table 2:** Main characteristics of all studies of *GSTT1* genotypes included in the meta-analysis.

**First author**	**Year**	**Country**	**Ethnicity**	**Subtype of acute leukemia**	**Case**	**Age** ^a^	**Sex** ^b^	**Control**	**Age** ^a^	**Sex** ^b^	**Case**		**Control**		**Control source**
											**Present**	**Null**	**Present**	**Null**	
Chen CL	1997	USA	White	ALL	163	N/A	85/78	213	18-60	111/102	140 (85.9)	23 (14.1)	181 (85.0)	32 (15.0)	Population
Chen CL	1997	USA	Black	ALL	34	N/A	22/12	203	18-60	103/100	22 (64.7)	12 (35.3)	154 (75.9)	49 (24.1)	Population
Krajinovic M	1999	Canada	White	ALL	176	1-21	N/A	274	N/A	N/A	148 (84.1)	28 (15.9)	227 (82.8)	47 (17.2)	Population
Woo MH	2000	USA	White	AML	40	N/A	N/A	160	N/A	N/A	33 (82.5)	7 (17.5)	138 (86.3)	22 (13.8)	Hospital
Woo MH	2000	USA	Black	AML	7	N/A	N/A	38	N/A	N/A	3 (42.9)	4 (57.1)	26 (68.4)	12 (31.6)	Hospital
Woo MH	2000	USA	Hispanic	AML	6	N/A	N/A	44	N/A	N/A	4 (66.7)	2 (33.3)	36 (81.8)	8 (18.2)	Hospital
Davies SM	2000	USA	White	AML	232	N/A	N/A	153	N/A	N/A	210 (90.5)	22 (9.5)	138 (90.2)	15 (9.8)	Population
Davies SM	2002	USA	White	ALL	616	N/A	N/A	532	N/A	N/A	520 (84.4)	96 (15.6)	445 (83.6)	87 (16.4)	Hospital
Davies SM	2002	USA	Black	ALL	35	N/A	N/A	201	N/A	N/A	29 (82.9)	6 (17.1)	145 (72.1)	56 (27.9)	Hospital
Alves S	2002	Portugal	White	ALL	47	N/A	N/A	102	N/A	N/A	38 (80.9)	9 (19.1)	76 (74.5)	26 (25.5)	Population
Balta G	2003	Turkey	White	ALL	139	0.58-17	96/48	185	0.58-17	120/65	110 (79.1)	29 (20.9)	143 (77.3)	42 (22.7)	Population
Balta G	2003	Turkey	White	ANLL	31	1-17	19/14	185	0.58-17	120/65	29 (93.5)	2 (6.5)	143 (77.3)	42 (22.7)	Population
Barnettee P	2004	USA	White	ALL	81	N/A	N/A	300	N/A	N/A	72 (88.9)	9 (11.1)	234 (78.0)	66 (22.0)	Population
Canalle R	2004	Brazil	White	ALL	113	0.33-18	73/40	221	18-58	159/62	88 (77.9)	25 (22.1)	178 (80.5)	43 (19.5)	Population
Joseph T	2004	India	Asian	ALL	118	0-14	77/41	118	0-14	77/41	101 (85.6)	17 (14.4)	108 (91.5)	10 (8.5)	Hospital
Wang J	2004	China	Asian	ALL	67	0.83-18	44/23	146	N/A	N/A	25 (37.3)	42 (62.7)	74 (50.7)	72 (49.3)	Population
Wang J	2004	China	Asian	AML	32	N/A	N/A	146	N/A	N/A	13 (40.6)	19 (59.4)	74 (50.3)	72 (49.3)	Population
Clavel J	2005	France	White	ALL	191	< 15	N/A	105	N/A	57/48	149 (78)	42 (22.0)	82 (78.1)	23 (21.9)	Hospital
Clavel J	2005	France	White	ANLL	28	< 15	N/A	105	N/A	57/48	22 (78.6)	6 (21.4)	82 (78.1)	23 (21.9)	Hospital
Pakakasama S	2005	Thailand	Asian	ALL	107	0.83-14.75	62/45	320	N/A	165/155	57 (53.3)	50 (46.7)	198 (61.9)	122 (38.1)	Population
Aydin-Sayitoglu M	2006	Turkey	White	ALL	119	N/A	N/A	140	16-59	73/67	90 (75.6)	29 (24.4)	111 (79.3)	29 (20.7)	Population
Aydin-Sayitoglu M	2006	Turkey	White	AML	44	N/A	N/A	140	16-59	73/67	38 (86.4)	6 (13.6)	111 (79.3)	29 (20.7)	Population
Pigullo S	2007	Italy	White	ALL	323	< 18	N/A	384	< 18	N/A	279 (86.4)	44 (13.6)	315 (82.0)	69 (18.0)	Hospital
Chan JY	2011	Indonesia	Asian	ALL	185	0.03-14	107/78	177	N/A	104/73	121 (65.4)	64 (34.6)	128 (72.3)	49 (27.7)	Population

^a^ range of age (year); ^b^ male/female; ALL, acute lymphoblastic leukemia; AML, acute myeloid leukemia; ANLL, acute non-lymphoblastic leukemia.

## Materials and Methods

### 1. Selection of published studies

Studies addressing the association between polymorphisms of *GSTM1* and *GSTT1* and the risk of childhood acute leukemia were identified by searching for articles in the PubMed and Chinese Biomedical Literature Database until 1 March 2013. Various combinations of the search terms ‘(*GSTM1* or *GSTT1*) and (polymorphism or polymorphisms) and childhood acute leukemia’ were used to screen for potentially relevant studies. Additional articles were also checked using the references cited in these publications. Articles that had data on the different types of childhood acute leukemia (e.g., acute lymphoblastic leukemia (ALL), acute myeloid leukemia (AML) and acute non-lymphoblastic leukemia (ANLL)) or different ethnic groups (e.g., Asians, Blacks and Whites) were treated as independent studies. Studies included in our meta-analysis had to meet all of the following criteria: (i) studied on human beings; (ii) in a case-control study design; and (iii) had detailed genotype frequency of cases and controls or could be calculated from the article text. In current study, data for meta-analysis were available from 17 articles (26 independent case-control studies), including 3252 cases and 5024 controls. 

### 2. Data extraction

Two independent researchers extracted raw data according to the inclusion criteria. If the two investigators generated different results, they would check the data again and have a discussion to make an agreement. If they could not reach an agreement, an expert was invited to the discussion. Data extracted from the selected articles included the first author’s name, year of publication, country of origin, ethnicity, subtype of acute leukemia, number of cases and controls, genotype frequency for cases and controls, and source of controls.

### 3. Statistical analysis

The risk of childhood acute leukemia that is associated with the polymorphisms of *GSTM1* and *GSTT1* genes were estimated for each study by odds ratio (OR), together with its 95% confidence interval (CI), respectively. Most studies evaluated *GSTM1* and *GSTT1* as presence/absence of gene deletion, so that meta-analysis of these polymorphisms were performed using a crude OR (null vs. present). A fixed-effect model using the Mantel-Haenszel method and a random-effects model using the DerSimonian and Laird method were used to combine values from studies. If the *P* value for heterogeneity was > 0.10 and *I* ^2^ < 50%, indicating an absence of heterogeneity between studies, we used the fixed-effect model to evaluate the summary ORs. In contrast, if the *P* value for heterogeneity was ≤ 0.10 or *I* ^2^ ≥ 50%, indicating a high extent of heterogeneity between studies, we used the random-effect model to evaluate the summary ORs.

Subgroup analyses were conducted by ethnicity (Asians, Blacks, and White), subtype of acute leukemia (ALL, AML and ANLL), number of cases and controls (

< 100 cases and <100 controls, ≥ 100 cases and ≥ 100 controls) and control source (Hospital-based, Population-based). Possible publication bias was tested by Begg’s funnel plot and Egger’s test. All analyses were performed using STATA software, version 9.2 (STATA Corp., College Station, TX).

## Results

### 1. Characteristics of Studies Analyzed

There were 117 articles relevant to searching strategy. The flow chart shown in Figure S1 summarizes the study selection process. Studies that had data on the different subtypes of acute leukemia or different ethnic groups were treated as independent studies. Thus, a total of 17 articles (26 independent case-control studies) including 3252 cases and 5024 controls were used in this meta-analysis. Publication dates ranged from 1997-2011. The characteristics of the selected studies are shown in Table 1 and Table 2. PRISMA checklist is shown in Table S1.

#### GSTM1 Polymorphism

A total of 26 studies were included in the meta-analysis with 3252 cases and 5024 controls. Cases consisted of 87.1% patients with ALL, 11.1% patients with AML and 1.8% patients with ANLL. Most of the controls (60.4%) were population-based participants. 

#### GSTT1 Polymorphism

Totally, 24 studies met the inclusion criteria and were selected in this meta-analysis with 2934 cases and 4592 controls. Cases consisted of 85.7% patients with ALL, 12.3% patients with AML and 2.0% patients with ANLL. Most of the controls (63.3%) were population-based participants. 

### 2. Meta-analysis of GSTM1 polymorphism and childhood acute leukemia

**Figure 1:**
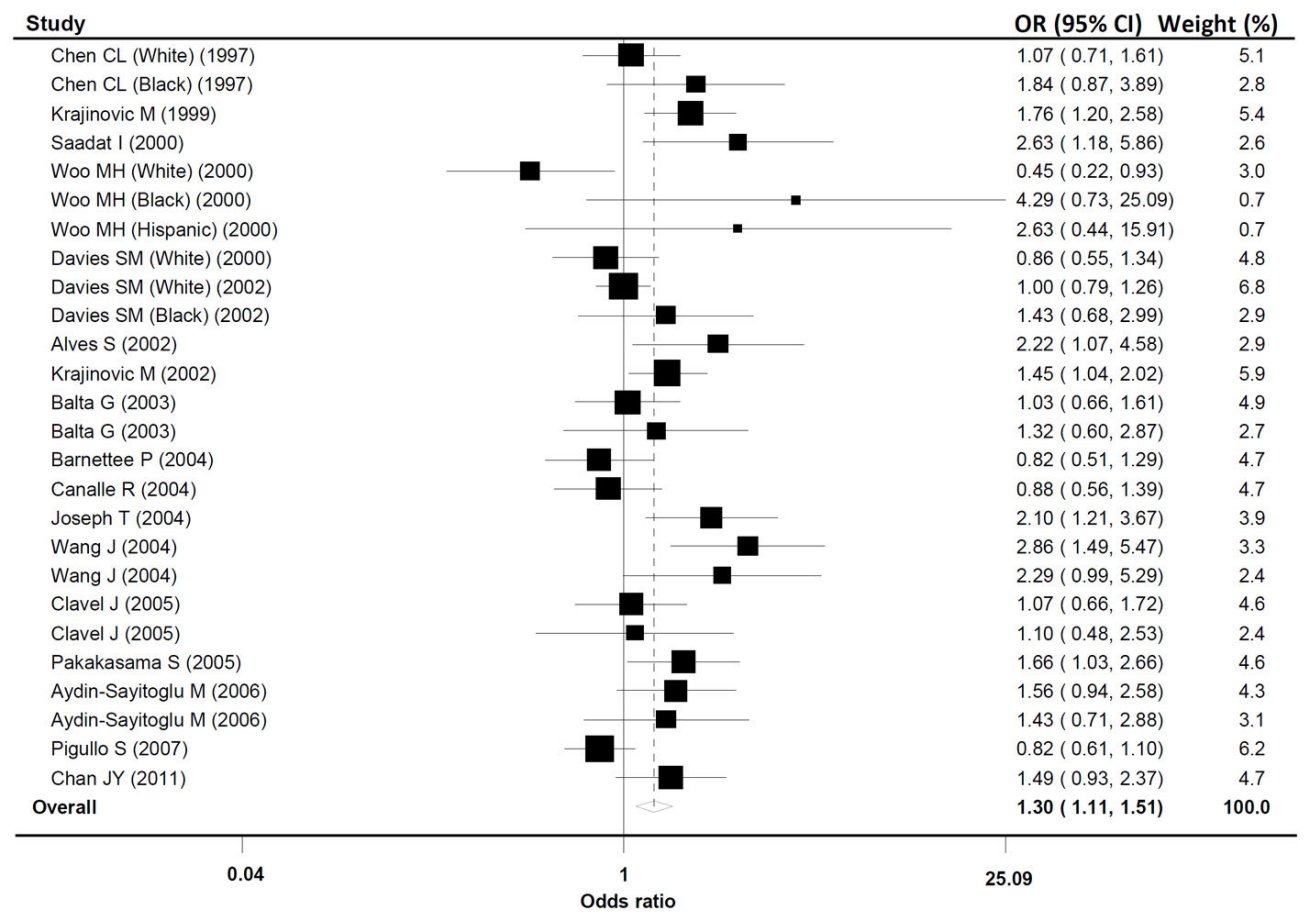
Forest plot of the *GSTM1* null polymorphism and childhood acute leukemia risk in overall analysis. Studies are plotted according to the last name of the first author. Horizontal lines represent 95% CI. Each square represents the OR point estimate and its size is proportional to the weight of the study. The diamond (and broken line) represents the overall summary estimate, with confidence interval given by its width. The unbroken vertical line is at the null value (OR = 1.0). CI, confidence interval; OR, odds ratio.

The evaluation of the association between *GSTM1* polymorphism and childhood acute leukemia risk is summarized in Table 3. A significantly elevated association between the null genotype of *GSTM1* polymorphism and childhood acute leukemia was found in all subjects (OR = 1.30; 95%CI, 1.11-1.51) (Figure 1). When stratified by ethnic groups, significantly elevated risks were observed in Asians (OR = 1.94; 95%CI, 1.53-2.46) and Blacks (OR = 1.76; 95%CI, 1.07-2.91) but not in Whites (OR = 1.09; 95%CI, 0.93-1.28). In the subgroup analysis by subtype of acute leukemia, significantly increased risks were observed in group of ALL (OR = 1.33; 95%CI, 1.13-1.58) but not in groups of AML and ANLL. Subgroup analysis based on the number of cases and controls showed that the increased risks were found in studies of ‘

< 100 cases and <100 controls’ (OR = 1.79; 95%CI, 1.21-2.64) and ‘≥ 100 cases and ≥ 100 controls’ (OR = 1.25; 95%CI, 1.02-1.55). Additionally, subgroup analysis by source of controls indicated that the null genotype has been associated with an increased risk of childhood acute leukemia in population-based studies (OR = 1.40; 95%CI, 1.15-1.69) but not in hospital-based studies (Table 3).

**Table 3:** Meta-analysis of case-control studies of *GSTM1* and *GSTT1* status and the risk of acute leukemia.

	**GSTM1**				**GSTT1**			
	**Studies**	**OR (95% CI)**	**P for heterogeneity**	***I*^2^** (%)	**Studies**	**OR (95% CI)**	**P for heterogeneity**	***I*^2^** (%)
**Total**	26	**1.30 (1.11-1.51**)**^a^**	< 0.001	55.5	24	1.02 (0.90-1.15)	0.130	25.1%
***Ethnic groups***								
**Asians**	6	**1.94 (1.53-2.46)**	0.577	0.0	5	**1.50 (1.17-1.93)**	0.962	0.0
**Blacks**	3	**1.76 (1.07-2.91)**	0.523	0.0	3	1.24 (0.48-3.19) ^a^	0.089	58.6
**Whites**	17	1.09 (0.93-1.28) ^a^	0.018	47.7	16	1.02 (0.90-1.15)	0.618	0.0
***Subtype of acute leukemia***								
**ALL**	20	**1.33 (1.13-1.58**)**^a^**	0.001	59.0	16	1.02 (0.90-1.17)	0.103	32.4
**AML**	4	1.24 (0.70-2.19) ^a^	0.018	63.2	6	1.14 (0.78-1.67)	0.343	6.5
**ANLL**	2	1.21 (0.69-2.14)	0.757	0.0	2	0.53 (0.13-2.17) ^a^	0.111	60.7
***Number of cases and controls***								
**< 100 cases and <100 controls**	3	**1.79 (1.21-2.64)**	0.882	0.0	2	2.61 (0.76-8.90)	0.844	0.0
**≥ 100 cases and ≥ 100 controls**	13	**1.25 (1.02-1.55**)**^a^**	0.011	53.6	12	1.04 (0.90-1.20)	0.537	0.0
***Source of controls***								
**Hospital-based**	10	1.14 (0.88-1.47) ^a^	0.009	58.9	9	0.94 (0.77-1.15)	0.370	7.9
**Population-based**	16	**1.40 (1.15-1.69**)**^a^**	0.015	48.9	15	1.01 (0.90-1.14)	0.106	32.8

^a^ Random-effects model was used when the *P*-value for heterogeneity test was ≤ 0.1 or *I* ^2^ ≥ 50%, otherwise the fixed-effect model was used. ALL, acute lymphoblastic leukemia; AML, acute myeloid leukemia; ANLL, acute non-lymphoblastic leukemia.

### 3. Meta-analysis of GSTT1 polymorphism and childhood acute leukemia

**Figure 2:**
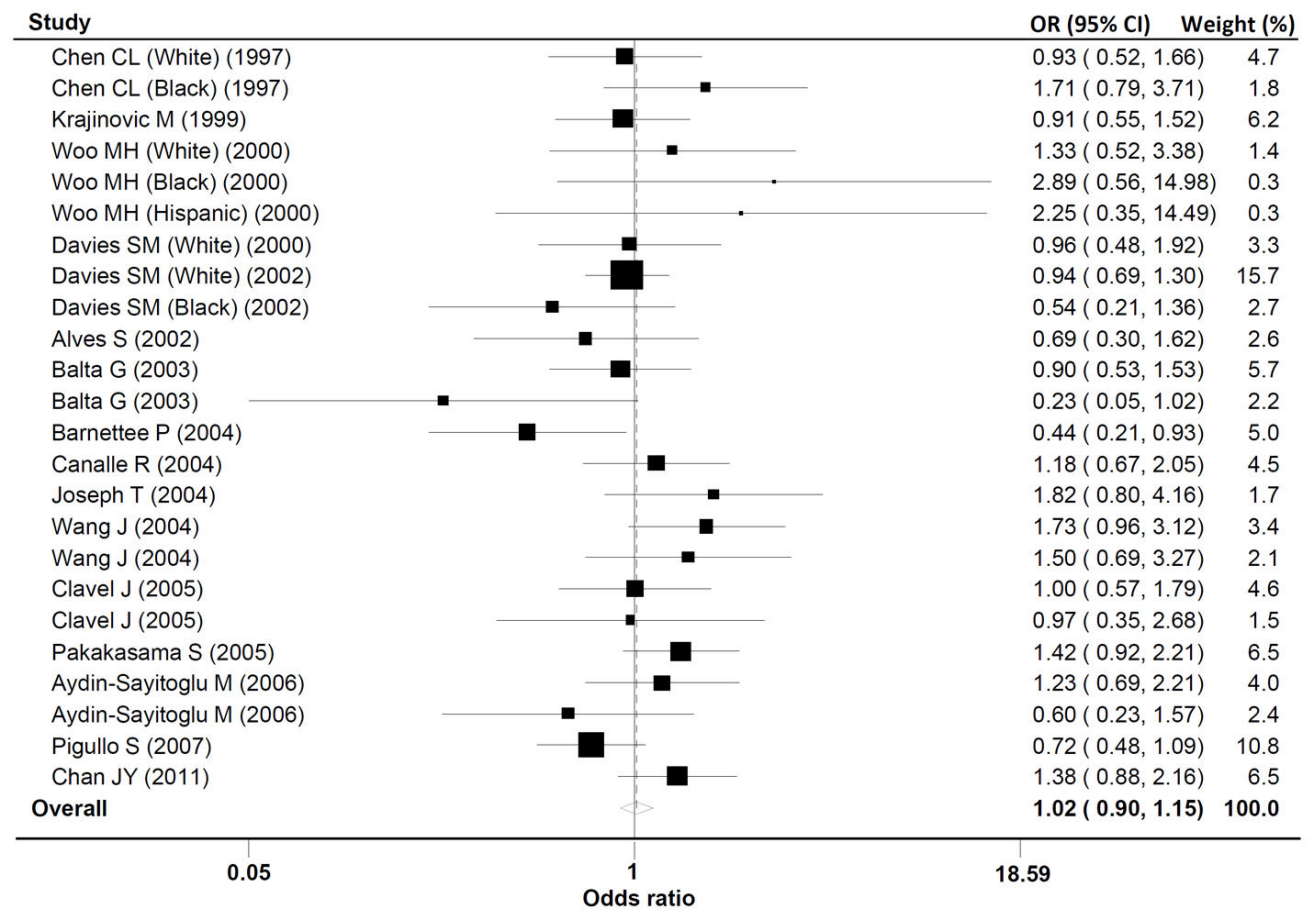
Forest plot of the *GSTT1* null polymorphism and childhood acute leukemia risk in overall analysis. Studies are plotted according to the last name of the first author. Horizontal lines represent 95% CI. Each square represents the OR point estimate and its size is proportional to the weight of the study. The diamond (and broken line) represents the overall summary estimate, with confidence interval given by its width. The unbroken vertical line is at the null value (OR = 1.0). CI, confidence interval; OR, odds ratio.

The evaluations of the association of *GSTT1* polymorphism and childhood acute leukemia are listed in Table 3. The null genotype of *GSTT1* polymorphism was associated with a significantly increased risk of childhood acute leukemia in Asians (OR = 1.50; 95%CI, 1.17-1.93) (Figure 2), while the association was not observed in the overall analysis and subgroup analysis according to subtype of acute leukemia, number of cases and controls, and source of controls (Table 3). 

### 4. Sensitive analysis

Sensitivity analyses were performed to determine whether modification of the inclusion criteria of the meta-analysis affected the final results. Although the sample size for cases and controls in 26 studies with a range from 6 to 616, the corresponding pooled ORs were not qualitatively altered with or without the study of small sample. Similarly, the sensitivity analysis indicated no other single study influenced the pooled ORs materially.

### 5. Publication Bias

Begg’s funnel plot and Egger’s test were performed to assess the publication bias of literatures. Figure S2 shows the funnel plot for the assessment of publication bias. For *GSTM1*, the shape of the funnel plot did not reveal any evidence of obvious asymmetry (*P* = 0.064) (Figure S2 A). However, the Egger’s test (*P* = 0.016) implied some evidence of publication bias. For *GSTT1*, both Begg’s test (*P* = 0.941) and Egger’s test (*P* = 0.991) did not suggest any evidence of publication bias (Figure S2 B). 

## Discussion

The present meta-analysis, including 3252 cases and 5024 controls from 26 case-control studies, exploring the association of *GSTM1* and *GSTT1* null polymorphisms with childhood acute leukemia risk. We demonstrated that the null polymorphism of *GSTM1* was associated with a significant increase in overall childhood acute leukemia risk, whereas the null polymorphism of *GSTT1* did not appear to have an overall influence on the susceptibility of childhood acute leukemia. Furthermore, in the stratified analyses of *GSTM1* null polymorphism, we found a significant influence on childhood acute leukemia risks in Asian and Black ethnic groups, ALL and population-based controls. However, we failed to find any significant relationships between *GSTT1* null polymorphism and childhood acute leukemia risk except in group of Asian. The association of the *GSTM1* null polymorphism but not the *GSTT1* polymorphism with childhood acute leukemia may be an indication of substrate specificity of *GSTM1* in metabolism of agents that are involved in the etiology of childhood acute leukemia.

 To the best of our knowledge, we conducted by far the largest and most comprehensive meta-analysis for quantitative analyses between the roles of the *GSTM1* and *GSTT1* polymorphisms and childhood acute leukemia risk. The *GSTM1* polymorphism is one of the most studied loci relating to childhood acute leukemia risk. The homozygous deletion resulting in functional loss of the GSTM1 enzyme has been implicated in the genesis of several cancers, including cervical neoplasia [15], colorectal cancer [34] and bladder cancer [35]. The present study suggests that the *GSTM1* null genotype is associated with a higher risk of childhood acute leukemia. In 2005, a meta-analysis by Ye et al. [16], had reported no overall association of polymorphisms of *GSTM1* and *GSTT1* with childhood ALL risk, including 4721 subjects (about half the size of our population of 8276). A recent meta-analysis of 15 published case-control studies on the effect of these polymorphisms and risk of childhood ALL was performed [36]. One observation in the latter study, i.e. *GSTM1* polymorphism but not *GSTT1* polymorphism was associated with the risk of childhood ALL [36], is similar to us. However, no association of these polymorphisms with ANLL were investigated in both meta-analysis studies [16,36] and no association of these polymorphisms with AML were investigated in the latter study [34]. Inclusion in our meta-analysis of few recent studies and data from CBM database could be the reason for the differences in the inference. 

Several factors must be considered in the design of a reliable case-control study in the future. Large sample size with adequate power is one of the most important factors. The choice of the control population is also considered to be a crucial factor because of the possible different exposure to environmental toxicants. Additionally, studies including information on the subtype of childhood acute leukemia are demanded to clarify the relationship between the GST polymorphisms and the subtypes of childhood acute leukemia.

There are some limitations should be acknowledged in this meta-analysis. Firstly, in the subgroup analyses of childhood acute leukemia, the number of AML and ANLL subgroups was relatively small, which don't have enough statistical power to explore the real association. Secondly, only three of the examined studies were performed in a Black population, so the ethnicity effect was not adequately investigated. Thirdly, our results were based on unadjusted estimates, while a more precise analysis should be conducted if all individual data was available, which would allow for the adjustment by other co-variants including age, gender, and environmental exposures. Fourthly, childhood acute leukemia is a multi-factorial disease that results from complex interactions between many genetic and environmental factors. It suggests that there will not be single gene or single environmental factor that has large effects on childhood acute leukemia susceptibility. In addition, as in most meta-analyses, publication bias must be considered because only published studies were included in the meta-analysis.

In conclusion, this meta-analysis showed that an increased risk of childhood acute leukemia is associated with the null polymorphism of *GSTM1*. It is necessary to conduct large sample studies using standardized unbiased genotyping methods, homogeneous patients with childhood acute leukemia and well matched controls. Additionally, more studies or complete case-control studies, especially stratified by different ethnic background, environmental exposure or other risk factors, should be performed to clarify possible roles of *GSTM1* and *GSTT1* null polymorphisms in the pathogenesis of childhood acute leukemia in the future.

## Supporting Information

Figure S1 **Flow chart of study identification.** Studies that had data on the different subtypes of acute leukemia (e.g., ALL, AML and ANLL) or different ethnic groups (e.g., Asians, Blacks and Whites) were treated as independent studies. Thus, a total of 26 studies were included in quantitative synthesis.
(TIF)

Figure S2 **Funnel plot analysis to detect publication bias.** Each point represents a separate study for the indicated association. Funnel plot for *GSTM1* (A) and *GSTT1* (B) null polymorphisms in overall analysis.
(TIF)

Table S1 **PRISMA Checklist.**
(DOC)
