# Supplementary figures and images for: GSTM1 and GSTT1 Null Polymorphisms and Childhood Acute Leukemia Risk: Evidence from 26 Case-Control Studies
Source: PLoS One. 2013 Oct 23;8(10):e78810. doi: 10.1371/journal.pone.0078810 (PMC3806859; doi:10.1371/journal.pone.0078810)

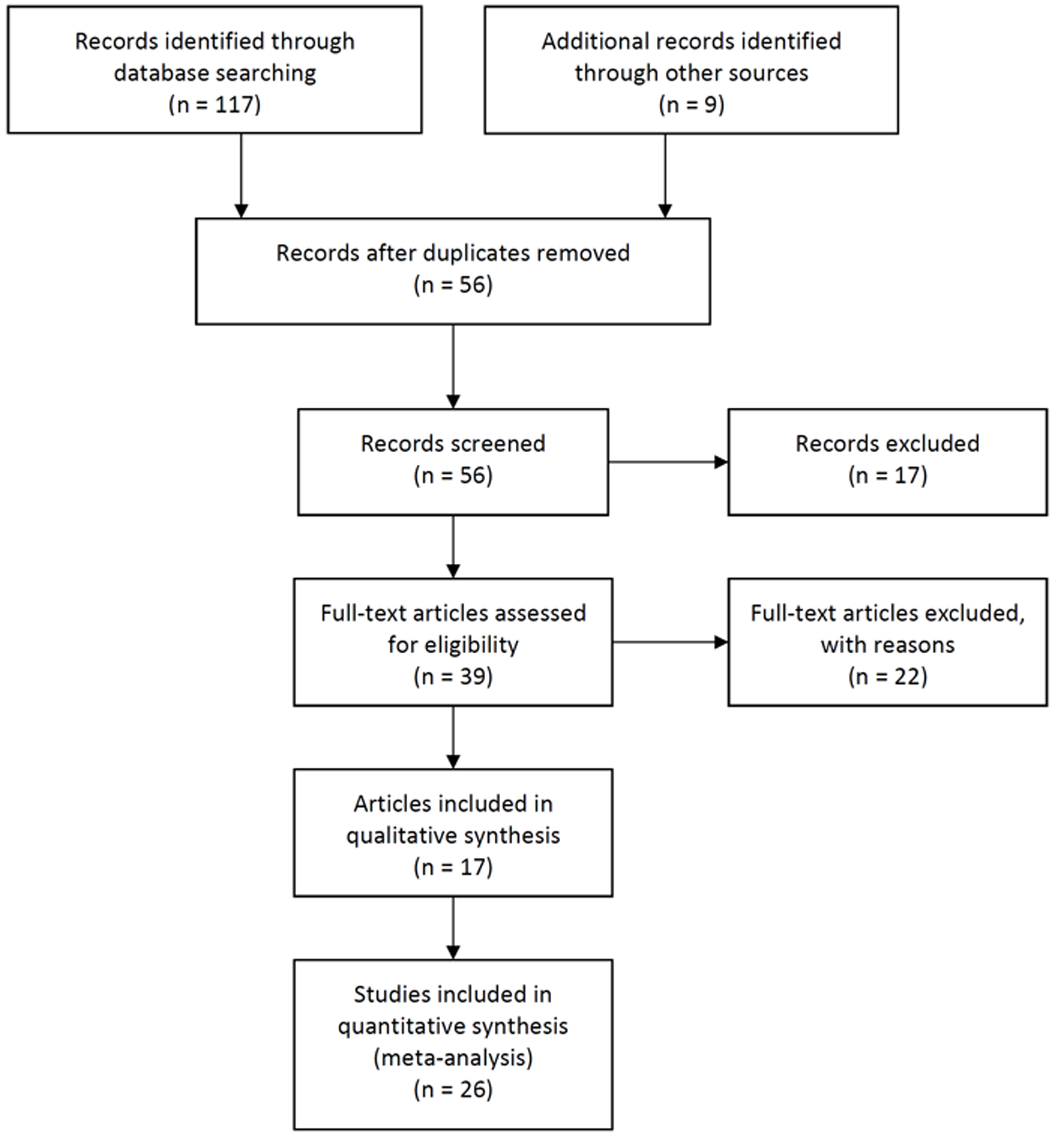

Supplement: Figure S1 — Flow chart of study identification. Studies that had data on the different subtypes of acute leukemia (e.g., ALL, AML and ANLL) or different ethnic groups (e.g., Asians, Blacks and Whites) were treated as independent studies. Thus, a total of 26 studies were included in quantitative synthesis. (TIF) [file pone.0078810.s001.tif]

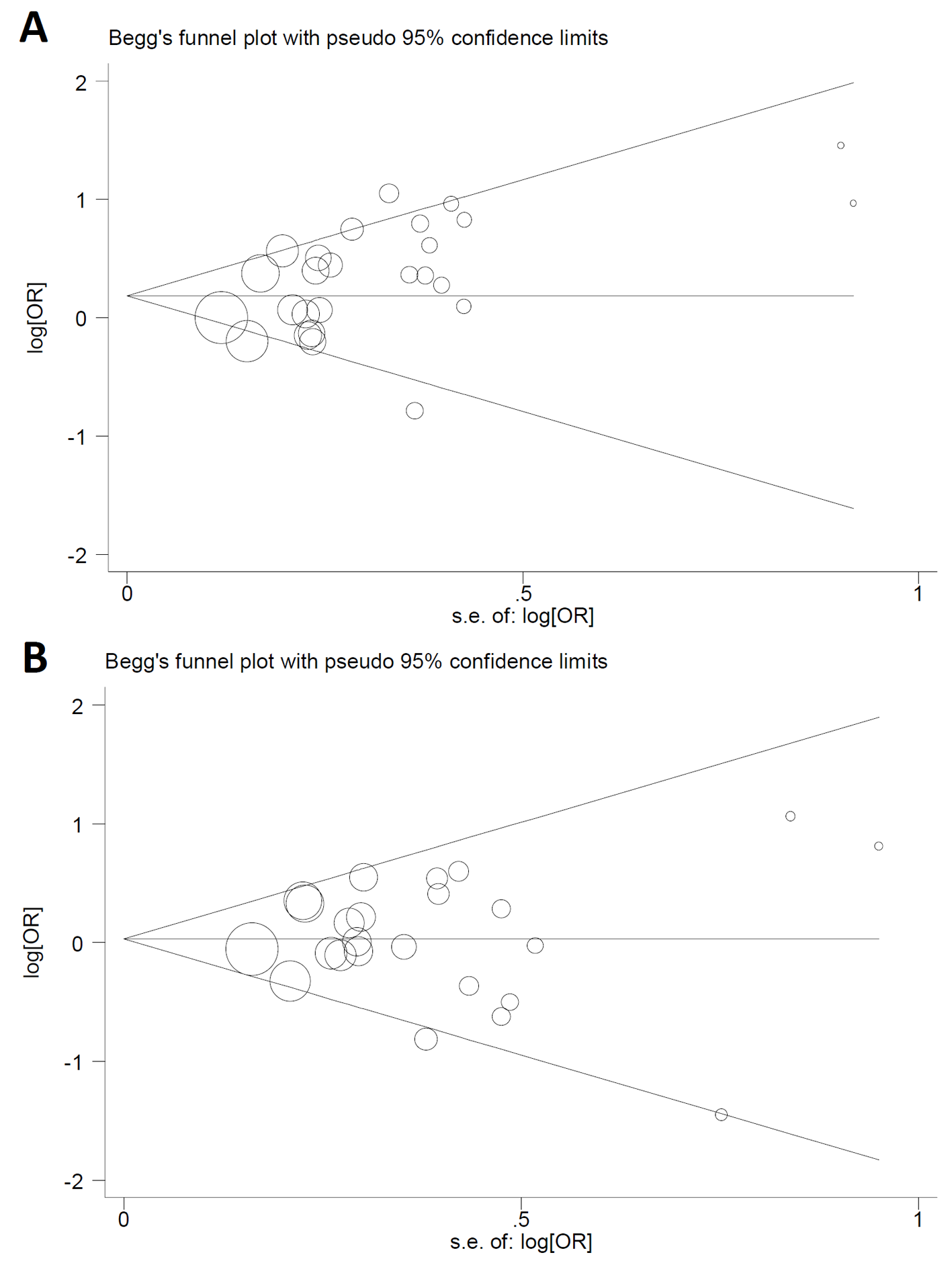

Supplement: Figure S2 — Funnel plot analysis to detect publication bias. Each point represents a separate study for the indicated association. Funnel plot for GSTM1 (A) and GSTT1 (B) null polymorphisms in overall analysis. (TIF) [file pone.0078810.s002.tif]
